# Host cell entry and neutralisation sensitivity of the SARS-CoV-2 XBB.1.16 lineage

**DOI:** 10.1038/s41423-023-01030-z

**Published:** 2023-05-08

**Authors:** Inga Nehlmeier, Amy Kempf, Prerna Arora, Anne Cossmann, Alexandra Dopfer-Jablonka, Metodi V. Stankov, Sebastian R. Schulz, Hans-Martin Jäck, Georg M. N. Behrens, Stefan Pöhlmann, Markus Hoffmann

**Affiliations:** 1 Infection Biology Unit, German Primate Center—Leibniz Institute for Primate Research, Kellnerweg 4, 37077 Göttingen, Germany; 2 Faculty of Biology and Psychology, Georg-August-University Göttingen, Wilhelmsplatz 1, 37073 Göttingen, Germany; 3 Department of Rheumatology and Immunology, Hannover Medical School, Carl-Neuberg-Straße 1, 30625 Hannover, Germany; 4 German Centre for Infection Research (DZIF), Partner Site Hannover-Braunschweig, Carl-Neuberg-Straße 1, 30625 Hannover, Germany; 5 Division of Molecular Immunology, Department of Internal Medicine 3, Friedrich-Alexander University of Erlangen-Nürnberg, Glückstraße 6, 91054 Erlangen, Germany; 6 Centre for Individualized Infection Medicine (CiiM), Feodor-Lynen-Straße 7, 30625 Hannover, Germany

**Keywords:** Infectious diseases, Immune evasion

Despite previous circulation of the highly transmissible and antibody evasive BA.2.75, BQ.1, XBB.1 and XBB.1.5 lineages, the share of infections caused by the SARS-CoV-2 lineage XBB.1.16 has gradually increased in India in early 2023, resulting in XBB.1.16 being the dominating SARS-CoV-2 lineage in India today. Since a similar trend may also take place in other countries and information on the biological properties of the XBB.1.16 lineage is scarce, we conducted a rapid assessment of the SARS-CoV-2 XBB.1.16 lineage with respect to its ability to enter cells and evade neutralisation by antibodies.

The newly emerged SARS-CoV-2 XBB.1.16 lineage (also dubbed as Arcturus), which harbours a unique combination of spike (S) protein mutations (Fig. 1a), was first described in India in January 2023 and rapidly became the dominating lineage (https://cov-spectrum.org/ [1]) (Fig. 1b). Here we performed a rapid assessment of the SARS-CoV-2 XBB.1.16 lineage regarding its ability to enter cells and evade neutralisation by antibodies using S protein-carrying pseudovirus particles (pp), which constitute a suitable model to study host cell entry of SARS-CoV-2 and its neutralisation [2]. For comparison, particles bearing the S protein of the ancestral B.1 lineage (B.1_pp_) or S proteins of Omicron sublineages BA.5 (BA.5_pp_), CH.1.1 (CH.1.1_pp_), XBB.1 (XBB.1_pp_), or XBB.1.5 (XBB.1.5_pp_) were used.

**Fig. 1:**
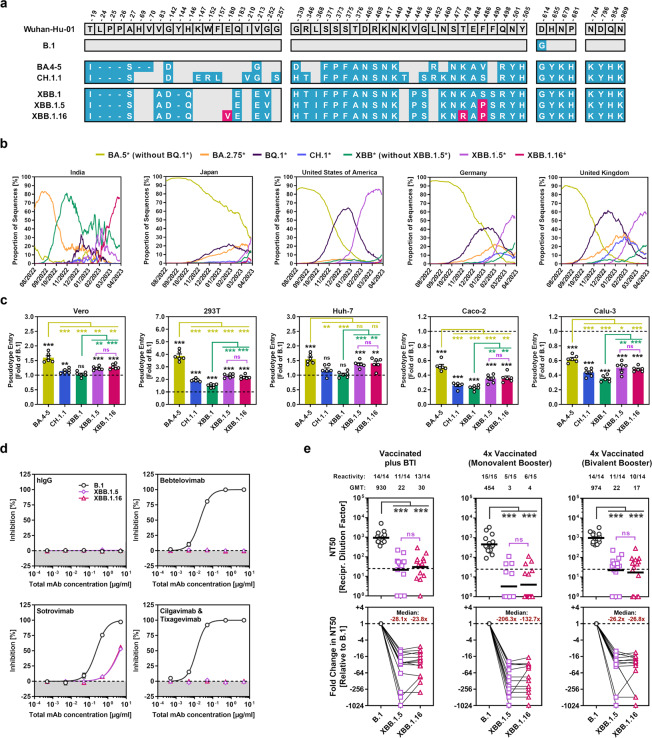
Host cell entry and neutralisation sensitivity of the SARS-CoV-2 XBB.1.16 lineage. **a** Mutations in the S proteins of SARS-CoV-2 lineages B.1, BA.4-5 (identical on protein level), CH.1.1, XBB.1, XBB.1.5 and XBB.1.16 compared to the S protein of the Wuhan-Hu-01 isolate. Unique mutations in the S proteins of XBB.1.5 and XBB.1.16 (compared to XBB.1) are highlighted in pink. **b** Relative frequency of SARS-CoV-2 lineages BA.5* (without BQ.1*), BA.2.75*, BQ.1*, CH.1*, XBB.1* (without XBB.1.5* and XBB.1.16), XBB.1.5*, and XBB.1.16* in selected countries. (Graphs are based on data obtained from https://cov-spectrum.org/). **c** Cell line tropism and entry efficiency of the SARS-CoV-2 XBB.1.16 lineage. Pseudovirus particles bearing the indicated S proteins were inoculated onto Vero (African green monkey, kidney), 293 T (human, kidney), Huh-7 (human, liver), Caco-2 (human, colon), and Calu-3 (human, lung) cells. Cell entry was analysed at 16–18 h postinoculation by measuring luciferase activity in cell lysates. Presented are the normalised mean data from six biological replicates (performed with four technical replicates) in which cell entry was normalised against particles carrying B.1 S protein (set as 1). Error bars represent the standard error of the mean (SEM). Statistical significance was assessed by two-tailed Student’s t-test with Welch correction (not significant [ns], *p* > 0.05; **p ≤ 0.01; ***p ≤ 0.001). Please see also Fig. S1. **d** Sensitivity of the SARS-CoV-2 XBB.1.16 lineage to neutralisation by monoclonal antibodies (mAb). Pseudotype particles harbouring the S protein of SARS-CoV-2 lineages B.1, XBB.1.5 or XBB.1.16 were preincubated with individual mAb or mAb cocktails for 30 min at room temperature, before being inoculated onto Vero cells (hIgG represents an unrelated control antibody). Pseudovirus entry was analysed at 16–18 h postinoculation and normalised against samples without mAb (= 0% inhibition). Data represent the mean of three biological replicates (performed with four technical replicates). Error bars indicate the SEM. **e** Sensitivity of the SARS-CoV-2 XBB.1.16 lineage to neutralisation by antibodies induced by vaccination or vaccination plus breakthrough infection. Pseudotype particles harbouring the S protein of SARS-CoV-2 lineage B.1, XBB.1.5, or XBB.1.16 were preincubated with plasma from (i) three- or four-times vaccinated individuals with breakthrough infection (BTI) between October 2022 and March 2023 in Germany (*n* = 14), (ii) four-times vaccinated individuals that received the monovalent BNT162b2/Comirnaty vaccine booster (*n* = 15), or (iii) four-times vaccinated individuals that received the bivalent BNT162b2/Comirnaty Original/Omicron BA.4-5 vaccine booster (*n* = 14). After an incubation period of 30 min at room temperature, samples were inoculated onto Vero cells. Pseudovirus entry was analysed at 16–18 h postinoculation, normalised against samples without plasma (0% inhibition), and the neutralising titre 50 (NT50, indicating the plasma dilution responsible for half-maximal inhibition) was calculated. Top panels: Presented are the geometric mean NT50 values (geometric mean titres, GMT) from a single biological replicate (conducted with four technical replicates). Numbers above the graphs represent reactivity rates (= proportion of plasma samples with detectable neutralising activity) and GMT. Statistical significance was assessed by Wilcoxon matched-pairs signed rank test (ns, *p* > 0.05; ***p ≤ 0.001). Please also see Table S1 and Figs. S2–S4. Bottom panels: Median fold change in neutralisation relative to B.1_pp_ (set as 1). Individual plasma are connected by lines

In line with expectations, BA.5_pp_ entered Vero (African green monkey, kidney), 293 T (human, kidney) and Huh-7 (human, liver) cells with higher efficiency compared to B.1_pp_ (1.6–3.9x higher, respectively), while entry into Caco-2 (human, colon) and Calu-3 (human, lung) cells was less efficient (1.6–1.9x reduced, compared to B.1_pp_) [3] (Fig. 1c). CH.1.1_pp_ and XBB.1_pp_ displayed comparable to slightly higher entry efficiency for Vero, 293 T and Huh-7 cells compared to B.1_pp_ (1.1x–1.9x higher, compared to B.1_pp_), while Caco-2 and Calu-3 cell entry was even less efficient as compared to BA.5_pp_ (2.3–3.8x reduced, compared to B.1_pp_) (Fig. 1c). In accordance with literature, cell entry of XBB.1.5_pp_ was generally increased compared to XBB.1_pp_ (1.2–1.5x higher, compared to XBB.1_pp_) [4] and the same observation was made for XBB.1.16_pp_ (1.3–1.6x higher, compared to XBB.1_pp_) (Fig. 1c).

As the mutations of XBB.1.16 might increase neutralisation evasion, we next investigated neutralisation sensitivity of XBB.1.16_pp_ to clinically used monoclonal antibodies (mAbs) as well as antibodies elicited upon vaccination, or vaccination plus breakthrough infection (BTI). With respect to mAb neutralisation, XBB.1.16_pp_ displayed partial resistance against Sotrovimab (effective concentration 50: 0.2 µg/ml [B.1] vs. 4.1 µg/ml [XBB.1.16]), and full resistance against Bebtelovimab, and a cocktail of Cilgavimab and Tixagevimab (Evusheld) (Fig. 1d), all of which is similar to what has been reported for XBB.1_pp_ [5] and XBB.1.5_pp_ (Fig. 1d) [4]. Finally, we analysed sensitivity of XBB.1.16_pp_ to neutralisation by vaccination- or vaccination plus BTI-induced antibodies. For the latter, we selected plasma from vaccinated individuals that became infected between October 2022 and March 2023 in Germany, a period in which sublineages of BA.5, BQ.1, BA.2.75, CH.1, and XBB.1.5 were abundant (Fig. 1b), since we did not have access to samples from individuals with proven XBB.1.5 infection. All plasma showed high neutralising activity against B.1_pp_, while the same plasma displayed strongly reduced neutralising activities against XBB.1.16_pp_ and XBB.1.5_pp_ (23.8–90.0x reduced and 26.2–132.7x reduced compared to B.1_pp_, respectively) (Fig. 1e). No differences in neutralisation sensitivity were observed between XBB.1.16_pp_ and XBB.1.5_pp_ (Fig. 1e).

Altogether, our data indicate that XBB.1.16_pp_ and XBB.1.5_pp_ display similar characteristics regarding cell line tropism, host cell entry efficiency and neutralisation evasion. This suggests that the recent increase in XBB.1.16 frequency in India may be linked to the immunisation background of the Indian population (e.g., high vaccination rate with vectored-adenovirus vaccines, pronounced circulation of BA.2.75 and early XBB.1 sublineages in the second half of 2022; Fig. 1b) or to specific properties of the XBB.1.16 lineage that could not be resolved within this study. Future studies are required to address these possibilities. For instance, it needs to be investigated whether XBB.1.16 has an advantage over XBB.1.5 when it comes to infection of individuals who have a history of infection by XBB.1.5 (or other XBB.1 sublineages).

## Supplementary information

Supplementary Material
