# Supplementary material for: Host cell entry and neutralisation sensitivity of the SARS-CoV-2 XBB.1.16 lineage
Source: Cell Mol Immunol. 2023 May 8;20(8):969–71. doi: 10.1038/s41423-023-01030-z (PMC10165563; doi:10.1038/s41423-023-01030-z)
Supplement: Supplementary file 1 — Supplementary Material [file 41423_2023_1030_MOESM1_ESM.docx]

**Host cell entry and neutralisation sensitivity of the SARS-CoV-2 XBB.1.16 lineage**

**Inga Nehlmeier^1^**^#^**, Amy Kempf^1,2^**^#^**, Prerna Arora^1,2^, Anne Cossmann^3^, Alexandra Dopfer-Jablonka^3,4^, Metodi V. Stankov^3^, Sebastian R. Schulz^5^, Hans-Martin Jäck^5^, Georg M. N. Behrens^3,4,6^, Stefan Pöhlmann^1,2^* Markus Hoffmann^1,2^***

^1^Infection Biology Unit, German Primate Center – Leibniz Institute for Primate Research, Kellnerweg 4, 37077 Göttingen, Germany.

^2^Faculty of Biology and Psychology, Georg-August-University Göttingen, Wilhelmsplatz 1, 37073 Göttingen, Germany.

^3^Department of Rheumatology and Immunology, Hannover Medical School, Carl-Neuberg-Straße 1, 30625 Hannover, Germany.

^4^German Centre for Infection Research (DZIF), partner site Hannover-Braunschweig, Carl-Neuberg-Straße 1, 30625 Hannover, Germany.

^5^Division of Molecular Immunology, Department of Internal Medicine 3, Friedrich-Alexander University of Erlangen-Nürnberg, Glückstraße 6, 91054 Erlangen, Germany.

^6^Centre for Individualized Infection Medicine (CiiM), Feodor-Lynen-Straße 7, 30625 Hannover, Germany.

^#^: These authors contributed equally

*: Corresponding authors: Markus Hoffmann ([mhoffmann@dpz.eu](mailto:mhoffmann@dpz.eu)) and Stefan Pöhlmann ([spoehlmann@dpz.eu](mailto:spoehlmann@dpz.eu))

# Methods

**Cell culture**

Vero (African green monkey kidney, female, kidney; CRL-1586, ATCC; RRID: CVCL 0574, kindly provided by Andrea Maisner), 293T (human, female, kidney; ACC-635, DSMZ; RRID: CVCL 0063) and Huh-7 (human, male, liver; JCRB Cat# JCRB0403; RRID: CVCL_0336, kindly provided by Thomas Pietschmann) cells were cultivated at 37 °C in a humidified atmosphere containing 5% CO_2_ using Dulbecco's modified Eagle medium (PAN-Biotech), supplemented with 10% fetal bovine serum (FBS, Biochrom), 1% penicillin/streptomycin solution (pen/strep, PAN-Biotech). Caco-2 (human, male, colon; HTB-37, ATCC, RRID: CVCL_0025) and Calu-3 (human, male, lung; HTB-55, ATCC; RRID: CVCL_0609, kindly provided by Stephan Ludwig) cells were cultured in minimum essential medium (Thermo Fisher Scientific) supplemented with 10% FBS, 1% pen/strep solution, 1% non-essential amino acid solution (PAA) and 1 mM sodium pyruvate (PAN-Biotech). All cell lines were validated by short tandem repeat (STR) analysis, amplification and sequencing of a cytochrome c oxidase gene fragment, microscopic examination, and/or according to their specific growth characteristics. Further, all cell lines were regularly screened for mycoplasma contamination. Transfection of 293T cells was carried out by calcium phosphate precipitation.

**Expression plasmids and sequence analysis**

Expression plasmids pCAGGS-DsRed (1), pCAGGS-VSV-G (2), pCG1-SARS-CoV-2 B.1 SΔ18 (codon-optimised, C-terminal truncation of 18 amino acid residues, GISAID Accession ID: EPI_ISL_425259) (3), pCG1-SARS-CoV-2 BA.5 SΔ18 (codon-optimised, C-terminal truncation of 18 amino acid residues, GISAID Accession ID: EPI_ISL_12029894) (4), XBB.1 (codon-optimised, C-terminal truncation of 18 amino acid residues, GISAID Accession ID: EPI_ISL_15384151) (5), and XBB.1.5 (codon-optimised, C-terminal truncation of 18 amino acid residues, GISAID Accession ID: EPI_ISL_16239158) (6) have been described before. In order to obtain the expression plasmid for SARS-CoV-2 XBB.1.16 SΔ18 (codon-optimised, C-terminal truncation of 18 amino acid residues, GISAID Accession ID: EPI_ISL_17237935), the respective mutations (E180V and K478R) were introduced into plasmid pCG1-SARS-CoV-2 XBB.1.5 SΔ18 by overlap-extension PCR with overlapping primers harbouring the respective mutations. Gibson assembly was employed to generate the expression plasmid for SARS-CoV-2 CH.1.1 SΔ18 (codon-optimised, C-terminal truncation of 18 amino acid residues, GISAID Accession ID: EPI_ISL_16292093). For this, overlapping DNA strings (synthesized by Thermo Fisher Scientific, sequences available upon request) were mixed with linearized (BamHI/XbaI-digested) pCG1 plasmid (a kind gift of Roberto Cattaneo, Mayo Clinic College of Medicine, Rochester, MN, USA) and GeneArt™ Gibson Assembly HiFi Master Mix (Thermo Fisher Scientific). The mixture was incubated for 45 min at 50 °C and subsequently transformed into one-shot OmniMAX 2 T1 competent *Escherichia coli* bacteria (Thermo Fisher Scientific). The following day, bacterial colonies were screened for presence of the insert by PCR, and positive clones were expanded for plasmid preparation. Integrity of S protein sequences was confirmed by Sanger sequencing using a commercial service (Microsynth SeqLab). The information on S protein sequences and lineage frequency was obtained from GISAID (Global Initiative on Sharing All Influenza Data) (<https://gisaid.org/>) and CoV-Spectrum (<https://cov-spectrum.org/>) databases.

**Pseudovirus particle production and cell entry**

Pseudovirus particles were produced according to a previously published protocol (7). For this, 293T cells were transfected to express the respective S protein, VSV-G, or DsRed (negative control). At 24h posttransfection, cells were inoculated with VSV-G-transcomplemented VSV*ΔG(FLuc) (kindly provided by Gert Zimmer) (8) and incubated for 1 h at 37 °C, before the inoculum was aspirated and cells were washed with PBS, and medium containing anti-VSV-G antibody (culture supernatant from I1-hybridoma cells; ATCC no. CRL-2700) was added (no antibody was added to cells expressing VSV-G). At 16-18 h postinoculation, cell culture supernatants were collected, clarified by centrifugation (4,000 x g, 10 min), and stored at -80 °C until further use. For experiments investigating the cell tropism and host cell entry efficiency of S protein-bearing pseudovirus particles, target cells were seeded into 96-well plates. After an incubation period of 24 h, cells were inoculated with equal volumes of pseudovirus particles and further incubated for 16-18 h. Next, the culture medium was aspirated and cells were lysed using PBS containing 0.5% Triton X-100 (Carl Roth). Following lysis for 30 min at room temperature, cell lysates were transferred into white 96-well plates, mixed with luciferase substrate (Beetle-Juice, PJK), and luminescence was recorded using a Hidex Sense plate luminometer (Hidex).

**Neutralisation assay**

Neutralisation assays were conducted as described earlier (9). Pseudovirus particles bearing SARS-CoV-2 S proteins were preincubated with different concentrations of monoclonal antibody (mAb; 5, 0.5, 0.05, 0.005, 0.0005 μg/ml) or different plasma dilutions (1:25, 1:100, 1:400, 1:1,600, 1:6,400) for 30 min at room temperature, and subsequently inoculated onto confluent monolayers of Vero cells that had been seeded into 96-well plates. Of note, for mAb cocktails each antibody was used at half the concentration to keep total antibody concentrations constant. Further, particles incubated in the absence of mAb/plasma served as controls. At 16-18 h postinoculation, luminescence was measured as described above. Neutralisation efficiency was calculated based on the relative inhibition of pseudovirus entry, for which pseudovirus particles incubated in the absence of mAb/plasma served as reference (= 0% inhibition). A non-linear regression model was used to calculate antibody concentrations (effective concentration 50, EC50) and plasma dilutions (neutralising titre 50, NT50) that caused half-maximal inhibition. Of note, plasma samples that yielded an NT50 value lower than 6.25 were considered negative and were assigned an NT50 value of 1.

**Ethics committee approval and enrolment of study participants**

The collection of plasma samples was performed after approval by the research ethics committee of the Institutional Review Board of Hannover Medical School (8973_BO_K_2020). All participants provided written informed consent prior to the use of plasma samples for research.

**Plasma samples**

A total of three cohorts were tested; cohort 1: three or four times vaccinated individuals that experienced a breakthrough infection (BTI) during between October 2022 and March 2023 in Germany (n = 14; median age = 38 years; male to female ratio 6:8); cohort 2 (V1/V2/V3/V4_monovalent_): individuals with a history of four vaccinations that received the monovalent BNT vaccine as fourth vaccination (n = 15; median age = 49.0 years; male to female ratio 5:10); cohort 3 (V1/V2/V3/V4_bivalent_): individuals with a history of four vaccinations that received the B.1/BA.4-5 bivalent BNT/Omicron BA.4-5 vaccine BNT vaccine as fourth vaccination (n = 14; median age = 53 years; male to female ratio 5:9).

Specific information can be found in the Appendix Table. SARS-CoV-2 infection-free status of cohorts 2 and 3 was confirmed by absence of anti-SARS-CoV-2 nucleocapsid protein (NCP) IgG using the Anti-SARS-CoV-2 NCP ELISA (IgG) (EUROIMMUN). A fraction of plasma samples was pre-screened for SARS-CoV-2 S1-specific IgG using the Anti-SARS-CoV-2-QuantiVac-ELISA (IgG) (EUROIMMUN) and all plasma samples were heat-inactivated (56 °C, 30 min) prior to neutralisation assays.

**Data analysis**

Data were analysed using Microsoft Excel (part of Microsoft Office Professional Plus, version 2016, Microsoft Corporation) and GraphPad Prism version 8.3.0 (GraphPad Software). Statistical significance was analysed by two-tailed Student’s t-test with Welch correction (cell line tropism) or Wilcoxon matched-pairs signed rank test (neutralisation). Effects were interpreted as statistically significant when p values of 0.05 or lower were obtained (ns [not significant], p > 0.05; *, p ≤ 0.05; **, p ≤ 0.01; ***, p ≤ 0.001).

**Limitations of the study**

Our study has limitations. First, although pseudovirus particles were shown to faithfully recapitulate SARS-CoV-2 host cell entry and its neutralisation, formal confirmation of our data with clinical SARS-CoV-2 isolates and primary cell cultures is required. Second, since the sample size for each cohort is relatively small, no investigation of differences in SARS-CoV-2 XBB.1.16 neutralisation as a result of biological factors (e.g. age or gender) is possible. Further, the small sample size precludes meaningful direct comparison of neutralising activity induced by booster vaccination with monovalent and bivalent vaccines. Third, since all plasma samples were collected within 3 months post vaccination/infection, we were not able to investigate SARS-CoV-2 XBB.1.16 neutralisation after extended time periods post vaccination/BTI. Fourth, as we did not have access to plasma samples of individuals with a history of proven (i.e., by sequencing of the infection SARS-CoV-2 lineage) XBB.1.5 or XBB.1 infection, we were not able to determine whether XBB.1.16 may have an advantage over XBB.1.5 when it comes to infection of these individuals.

# Supplementary references

1. Hoffmann M, Kleine-Weber H, Schroeder S, Kruger N, Herrler T, Erichsen S, et al. SARS-CoV-2 Cell Entry Depends on ACE2 and TMPRSS2 and Is Blocked by a Clinically Proven Protease Inhibitor. Cell. 2020;181(2):271-80 e8.

2. Brinkmann C, Hoffmann M, Lubke A, Nehlmeier I, Kramer-Kuhl A, Winkler M, et al. The glycoprotein of vesicular stomatitis virus promotes release of virus-like particles from tetherin-positive cells. PLoS One. 2017;12(12):e0189073.

3. Hoffmann M, Arora P, Gross R, Seidel A, Hornich BF, Hahn AS, et al. SARS-CoV-2 variants B.1.351 and P.1 escape from neutralizing antibodies. Cell. 2021;184(9):2384-93 e12.

4. Arora P, Kempf A, Nehlmeier I, Schulz SR, Cossmann A, Stankov MV, et al. Augmented neutralisation resistance of emerging omicron subvariants BA.2.12.1, BA.4, and BA.5. The Lancet Infectious diseases. 2022;22(8):1117-8.

5. Arora P, Cossmann A, Schulz SR, Ramos GM, Stankov MV, Jack HM, et al. Neutralisation sensitivity of the SARS-CoV-2 XBB.1 lineage. Lancet Infect Dis. 2023;23(2):147-8.

6. Hoffmann M, Arora P, Nehlmeier I, Kempf A, Cossmann A, Schulz SR, et al. Profound neutralization evasion and augmented host cell entry are hallmarks of the fast-spreading SARS-CoV-2 lineage XBB.1.5. Cell Mol Immunol. 2023;20(4):419-22.

7. Kleine-Weber H, Elzayat MT, Wang L, Graham BS, Muller MA, Drosten C, et al. Mutations in the Spike Protein of Middle East Respiratory Syndrome Coronavirus Transmitted in Korea Increase Resistance to Antibody-Mediated Neutralization. J Virol. 2019;93(2).

8. Berger Rentsch M, Zimmer G. A vesicular stomatitis virus replicon-based bioassay for the rapid and sensitive determination of multi-species type I interferon. PLoS One. 2011;6(10):e25858.

9. Arora P, Zhang L, Kruger N, Rocha C, Sidarovich A, Schulz S, et al. SARS-CoV-2 Omicron sublineages show comparable cell entry but differential neutralization by therapeutic antibodies. Cell host & microbe. 2022;30(8):1103-11 e6.

# Supplementary Tables

**Table S1: Plasma information**

| **Cohort** | **ID** | **Gender** | **Age**  **(years)** | **V1/V2/V3** | **V4** | **BTI** | **Time between sampling and last vaccination (days)** | **Time between sampling and positive PCR (days)** | **Anti-SARS-CoV-2 S1 IgG**  **(BAU/ml)** |
| --- | --- | --- | --- | --- | --- | --- | --- | --- | --- |
| **1** | **9618** | **Female** | **50** | **no information** | **yes (no information)** | **yes** | **229** | **4** | **4,921** |
| **1** | **9717** | **Male** | **42** | **no information** | **yes (no information)** | **yes** | **36** | **28** | **13,935** |
| **1** | **9783** | **Female** | **40** | **no information** | **yes (no information)** | **yes** | **22** | **15** | **4,049** |
| **1** | **9786** | **Male** | **21** | **no information** | **no** | **yes** | **392** | **17** | **5,933** |
| **1** | **9798** | **Male** | **54** | **no information** | **yes (no information)** | **yes** | **56** | **40** | **7,002** |
| **1** | **9805** | **Female** | **62** | **no information** | **no** | **yes** | **414** | **63** | **3,949** |
| **1** | **9826** | **Female** | **38** | **no information** | **no** | **yes** | **452** | **17** | **2,946** |
| **1** | **9833** | **Female** | **22** | **no information** | **no** | **yes** | **473** | **14** | **not analysed** |
| **1** | **9834** | **Male** | **44** | **no information** | **no** | **yes** | **510** | **23** | **not analysed** |
| **1** | **9835** | **Male** | **32** | **no information** | **yes (no information)** | **yes** | **125** | **37** | **not analysed** |
| **1** | **9836** | **Female** | **30** | **no information** | **yes (no information)** | **yes** | **152** | **39** | **not analysed** |
| **1** | **9837** | **Male** | **32** | **no information** | **no** | **yes** | **no information** | **16** | **not analysed** |
| **1** | **9838** | **Female** | **30** | **no information** | **yes (no information)** | **yes** | **365** | **16** | **not analysed** |
| **1** | **9839** | **Female** | **no information** | **no information** | **no** | **yes** | **476** | **37** | **not analysed** |
| **2** | **8220** | **Female** | **57** | **AZ/AZ/MOD** | **yes**  **(BNT)** | **no** | **37** | **n.a.** | **2,031** |
| **2** | **8221** | **Male** | **48** | **AZ/AZ/BNT** | **yes**  **(BNT)** | **no** | **30** | **n.a.** | **1,826** |
| **2** | **8380** | **Male** | **49** | **AZ/BNT/BNT** | **yes**  **(BNT)** | **no** | **42** | **n.a.** | **2,022** |
| **2** | **8383** | **Male** | **44** | **BNT/BNT/BNT** | **yes**  **(BNT)** | **no** | **37** | **n.a.** | **8,018** |
| **2** | **8391** | **Female** | **38** | **BNT/BNT/BNT** | **yes**  **(BNT)** | **no** | **40** | **n.a.** | **2,341** |
| **2** | **8808** | **Female** | **59** | **AZ/BNT/BNT** | **yes**  **(BNT)** | **no** | **7** | **n.a.** | **4,709** |
| **2** | **8830** | **Male** | **65** | **AZ/AZ/BNT** | **yes**  **(BNT)** | **no** | **9** | **n.a.** | **1,931** |
| **2** | **8864** | **Female** | **49** | **BNT/BNT/BNT** | **yes**  **(BNT)** | **no** | **46** | **n.a.** | **7,836** |
| **2** | **9076** | **Female** | **49** | **BNT/BNT/BNT** | **yes**  **(BNT)** | **no** | **74** | **n.a.** | **1,704** |
| **2** | **9170** | **Female** | **62** | **BNT/BNT/BNT** | **yes**  **(BNT)** | **no** | **71** | **n.a.** | **5,620** |
| **2** | **9182** | **Female** | **60** | **BNT/BNT/BNT** | **yes**  **(BNT)** | **no** | **83** | **n.a.** | **3,831** |
| **2** | **9292** | **Female** | **31** | **BNT/BNT/BNT** | **yes**  **(BNT)** | **no** | **51** | **n.a.** | **5,424** |
| **2** | **9310** | **Male** | **53** | **BNT/BNT/BNT** | **yes**  **(BNT)** | **no** | **2** | **n.a.** | **4,990** |
| **2** | **9351** | **Female** | **27** | **BNT/BNT/BNT** | **yes**  **(BNT)** | **no** | **29** | **n.a.** | **11,368** |
| **2** | **9357** | **Female** | **58** | **BNT/BNT/BNT** | **yes**  **(BNT)** | **no** | **33** | **n.a.** | **1,668** |
| **3** | **9387** | **Female** | **59** | **BNT/BNT/BNT** | **yes**  **(BNT_bivalent_)** | **no** | **27** | **n.a.** | **6,161** |
| **3** | **9445** | **Male** | **65** | **AZ/AZ/BNT** | **yes**  **(BNT_bivalent_)** | **no** | **27** | **n.a.** | **7,177** |
| **3** | **9446** | **Female** | **61** | **BNT/BNT/BNT** | **yes**  **(BNT_bivalent_)** | **no** | **27** | **n.a.** | **5,104** |
| **3** | **9448** | **Female** | **56** | **BNT/BNT/BNT** | **yes**  **(BNT_bivalent_)** | **no** | **11** | **n.a.** | **7,945** |
| **3** | **9452** | **Male** | **44** | **no information** | **yes**  **(BNT_bivalent_)** | **no** | **27** | **n.a.** | **12,697** |
| **3** | **9476** | **Female** | **51** | **no information** | **yes**  **(BNT_bivalent_)** | **no** | **33** | **n.a.** | **5429** |
| **3** | **9481** | **Female** | **58** | **no information** | **yes**  **(BNT_bivalent_)** | **no** | **23** | **n.a.** | **8758** |
| **3** | **9484** | **Male** | **45** | **no information** | **yes**  **(BNT_bivalent_)** | **no** | **3** | **n.a.** | **7,828** |
| **3** | **9486** | **Female** | **52** | **no information** | **yes**  **(BNT_bivalent_)** | **no** | **23** | **n.a.** | **8,519** |
| **3** | **9488** | **Male** | **54** | **no information** | **yes**  **(BNT_bivalent_)** | **no** | **26** | **n.a.** | **2,644** |
| **3** | **9491** | **Female** | **46** | **no information** | **yes**  **(BNT_bivalent_)** | **no** | **33** | **n.a.** | **4,773** |
| **3** | **9493** | **Female** | **61** | **no information** | **yes**  **(BNT_bivalent_)** | **no** | **28** | **n.a.** | **4,819** |
| **3** | **9494** | **Female** | **50** | **no information** | **yes**  **(BNT_bivalent_)** | **no** | **33** | **n.a.** | **3,610** |
| **3** | **9496** | **Male** | **40** | **BNT/BNT/MOD** | **yes**  **(BNT_bivalent_)** | **no** | **33** | **n.a.** | **8,194** |

Cohort 1: Three or four times vaccinated plus BTI; Cohort 2: Four times vaccinated (monovalent booster); Cohort 3: Four times vaccinated (bivalent booster).

^a^: SARS-CoV-2 infection-free status of cohorts 2 and 3 was confirmed by ELISA (= anti-NCP IgG-negative).

^b^: Anti-SARS-CoV-2 S1 IgG titres were determined against ancestral SARS-CoV-2.

Abbreviations: V#, vaccination; AZ, AZD1222/Vaxzevria; BNT, BNT162b2/Comirnaty; MOD, mRNA-1273/Spikevax; BNT_bivalent_, Comirnaty Original/Omicron BA.4-5; BAU, binding antibody units; BTI, breakthrough infection; IgG, immunoglobulin G; n.a., not applicable.

# Supplementary figures


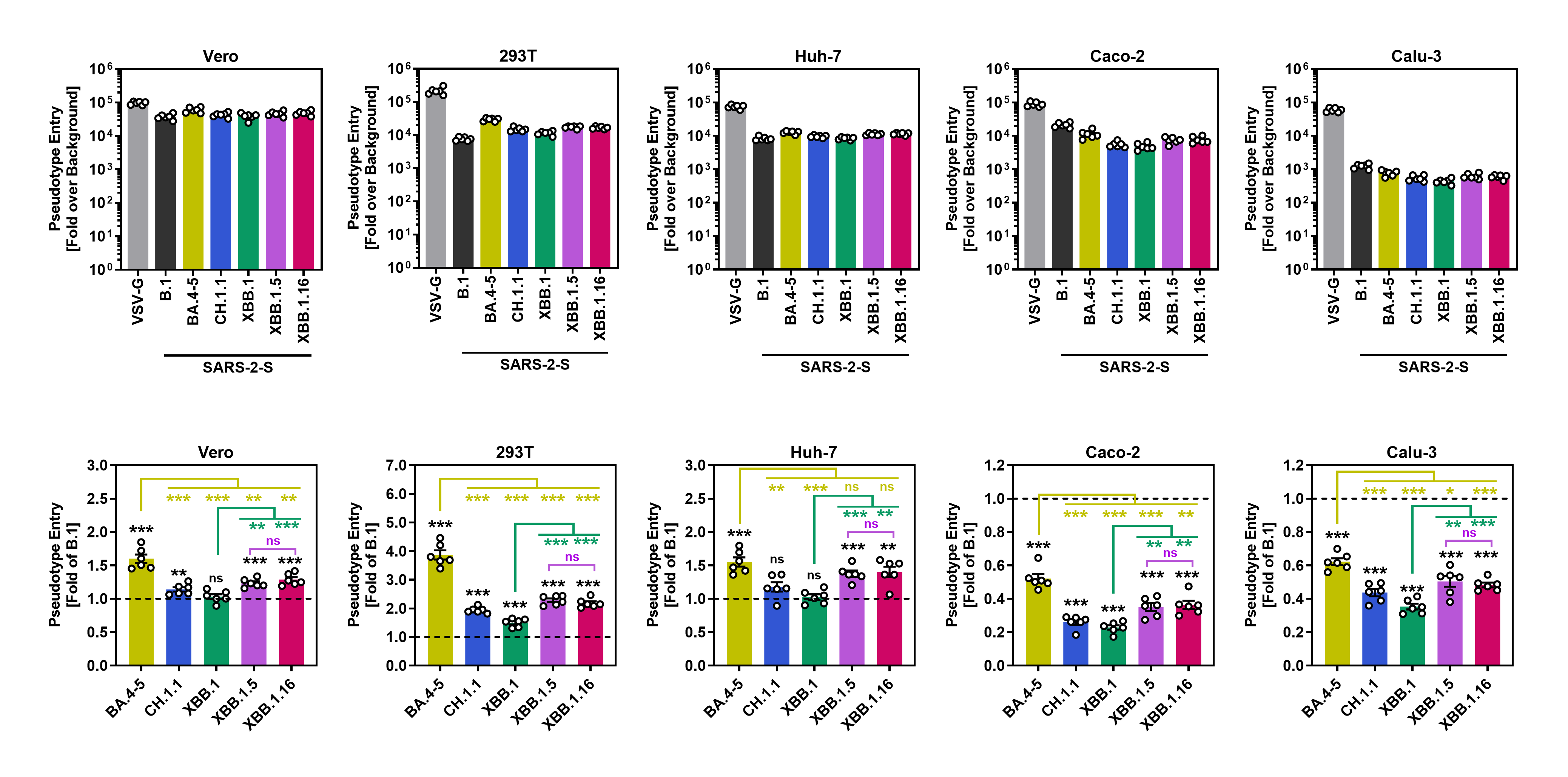


**Figure S1: Cell line tropism and entry efficiency of particles bearing the S protein of the SARS-CoV-2 XBB.1.16 lineage.**

Pseudovirus particles bearing the indicated S proteins or vesicular stomatitis virus glycoprotein (VSV-G) were inoculated onto Vero (African green monkey, kidney), 293T (human, kidney), Huh-7 (human, liver), Caco-2 (human, colon), and Calu-3 (human, lung) cells. Cell entry was analysed at 16-18 h postinoculation by measuring luciferase activity in cell lysates. Presented are the normalised mean data from six biological replicates (performed with four technical replicates) in which cell entry was normalised against particles bearing no viral glycoprotein (set as 1). Error bars represent the standard error of the mean (SEM).


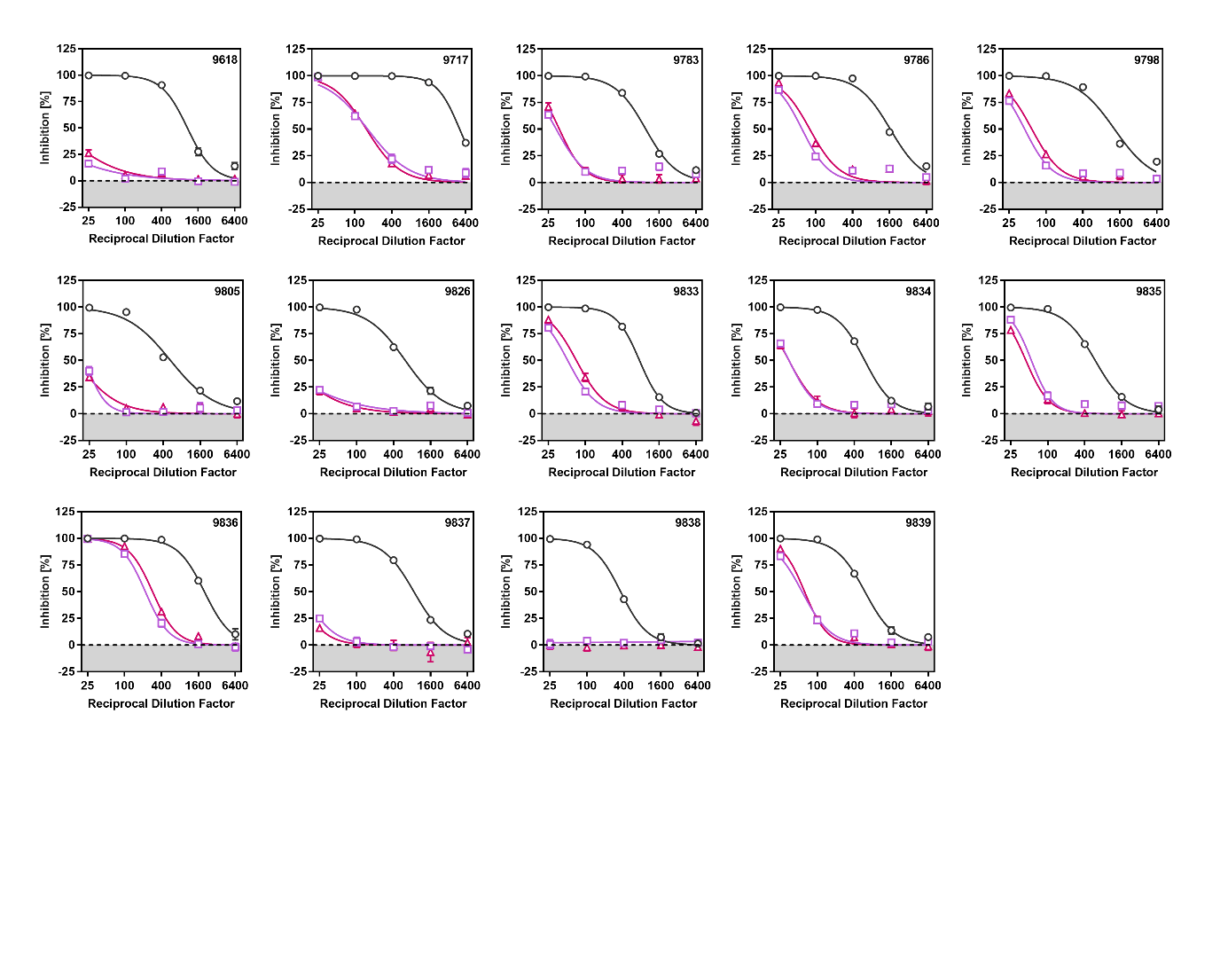


**Figure S2: Individual neutralisation data for cohort 1: Vaccinated plus BTI.**

Individual plasma neutralisation data for pseudoviruses carrying the S protein of SARS-CoV-2 lineages B.1 (dark grey), XBB.1.5 (purple), or XBB.1.16 (pink).

**
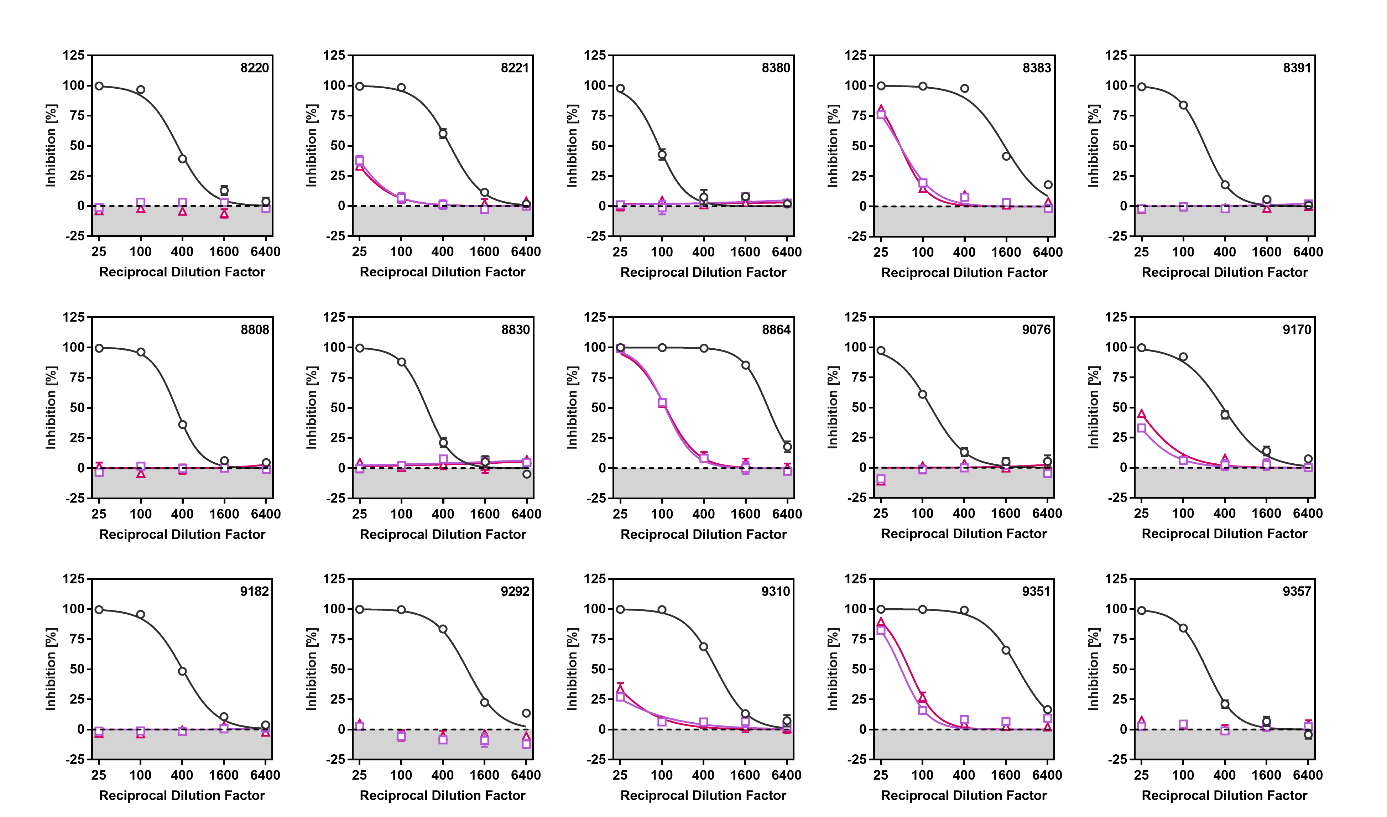
**

**Figure S3: Individual neutralisation data for cohort 2: 4x Vaccinated (Monovalent Booster).**

Individual plasma neutralisation data for pseudoviruses carrying the S protein of SARS-CoV-2 lineages B.1 (dark grey), XBB.1.5 (purple), or XBB.1.16 (pink).


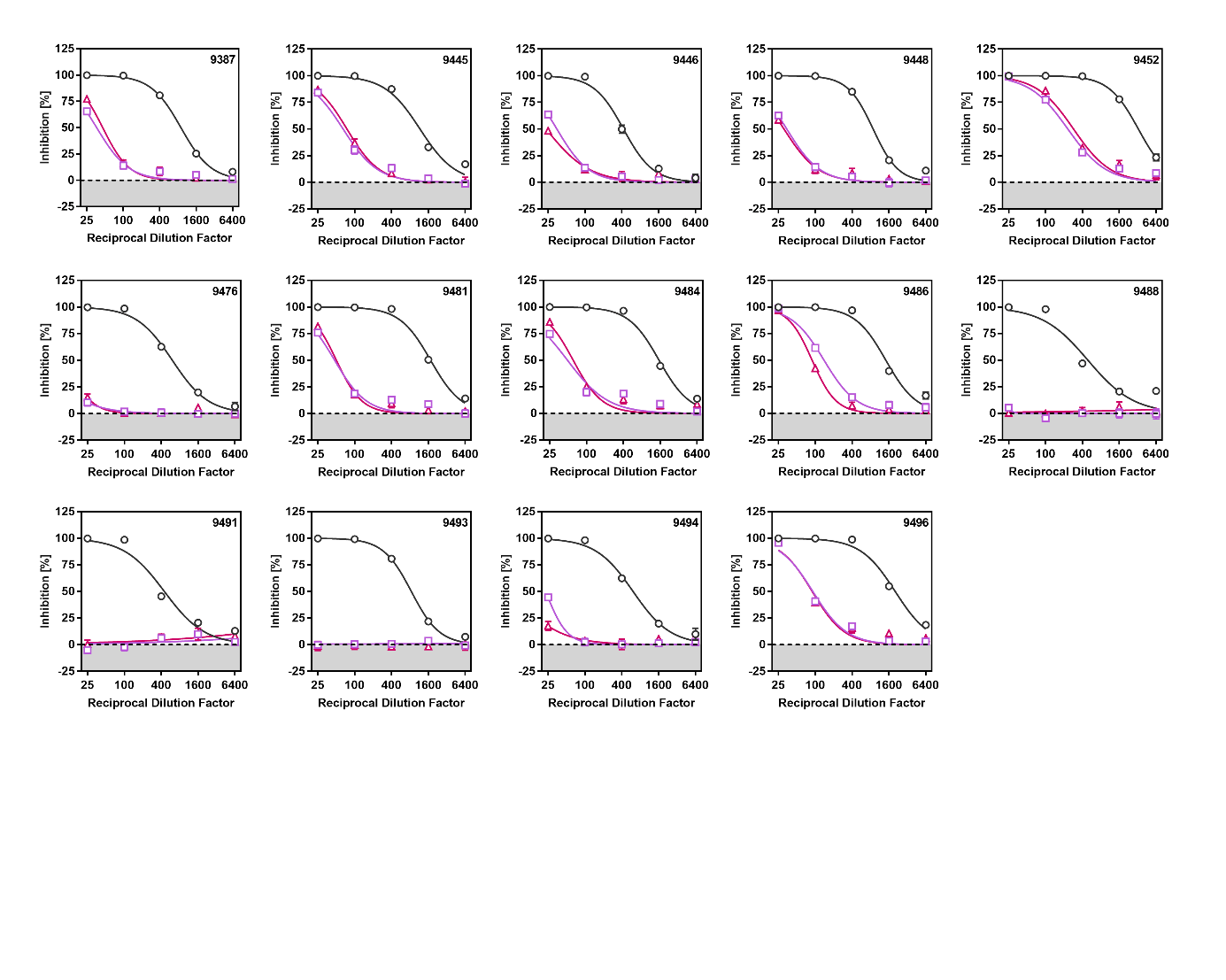


**Figure S4: Individual neutralisation data for cohort 2: 4x Vaccinated (Monovalent Booster).**

Individual plasma neutralisation data for pseudoviruses carrying the S protein of SARS-CoV-2 lineages B.1 (dark grey), XBB.1.5 (purple), or XBB.1.16 (pink).
